# Supplementary figures and images for: Mammalian orthoreovirus can exit cells in extracellular vesicles
Source: PLoS Pathog. 2024 Jan 11;20(1):e1011637. doi: 10.1371/journal.ppat.1011637 (PMC10807757; doi:10.1371/journal.ppat.1011637)

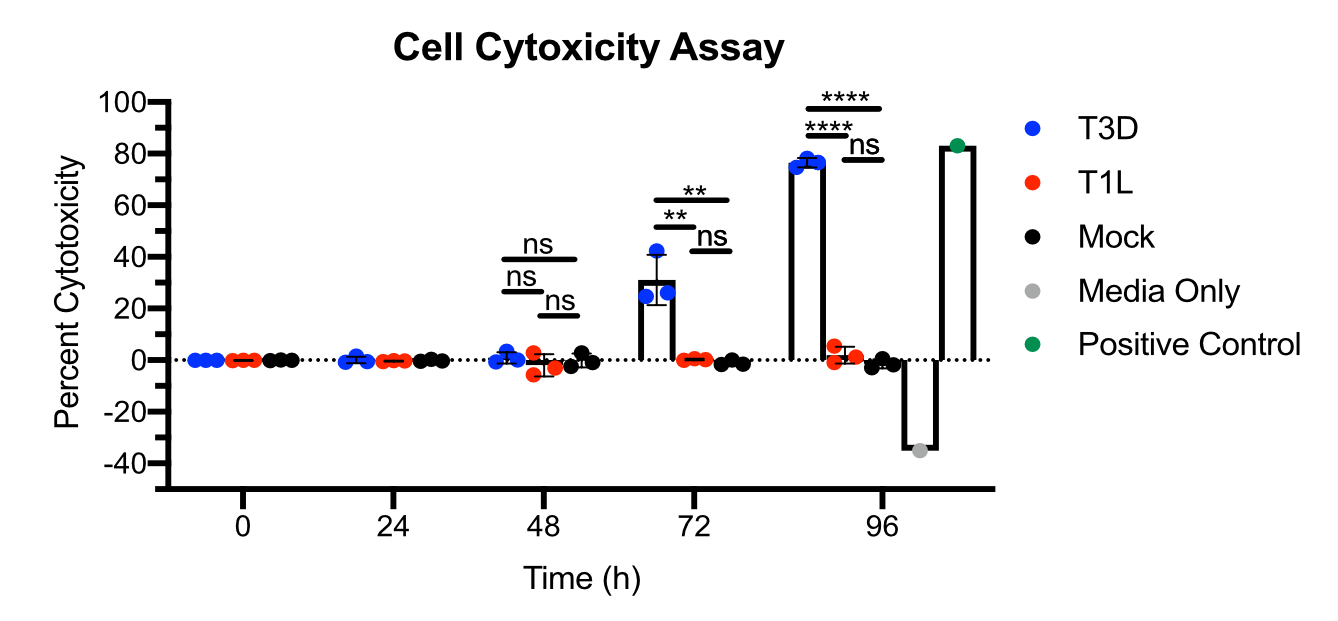

Supplement: S1 Fig — L cells were adsorbed with medium (mock) or with three individual clones of T1L or T3D reovirus at an MOI of 1 PFU/cell. Cell membrane disruption was quantified for T1L-, T3D-, and mock-infected cells every 24 h for 96 h using an LDH assay. A medium-only negative control and a kit-specific positive control quantified in triplicate at 96 h are shown. Error bars indicate SD. n = 3. **, P < 0.01; ****, P < 0.0001 by one-way ANOVA with Tukey’s multiple comparisons. (TIF) [file ppat.1011637.s001.tif]

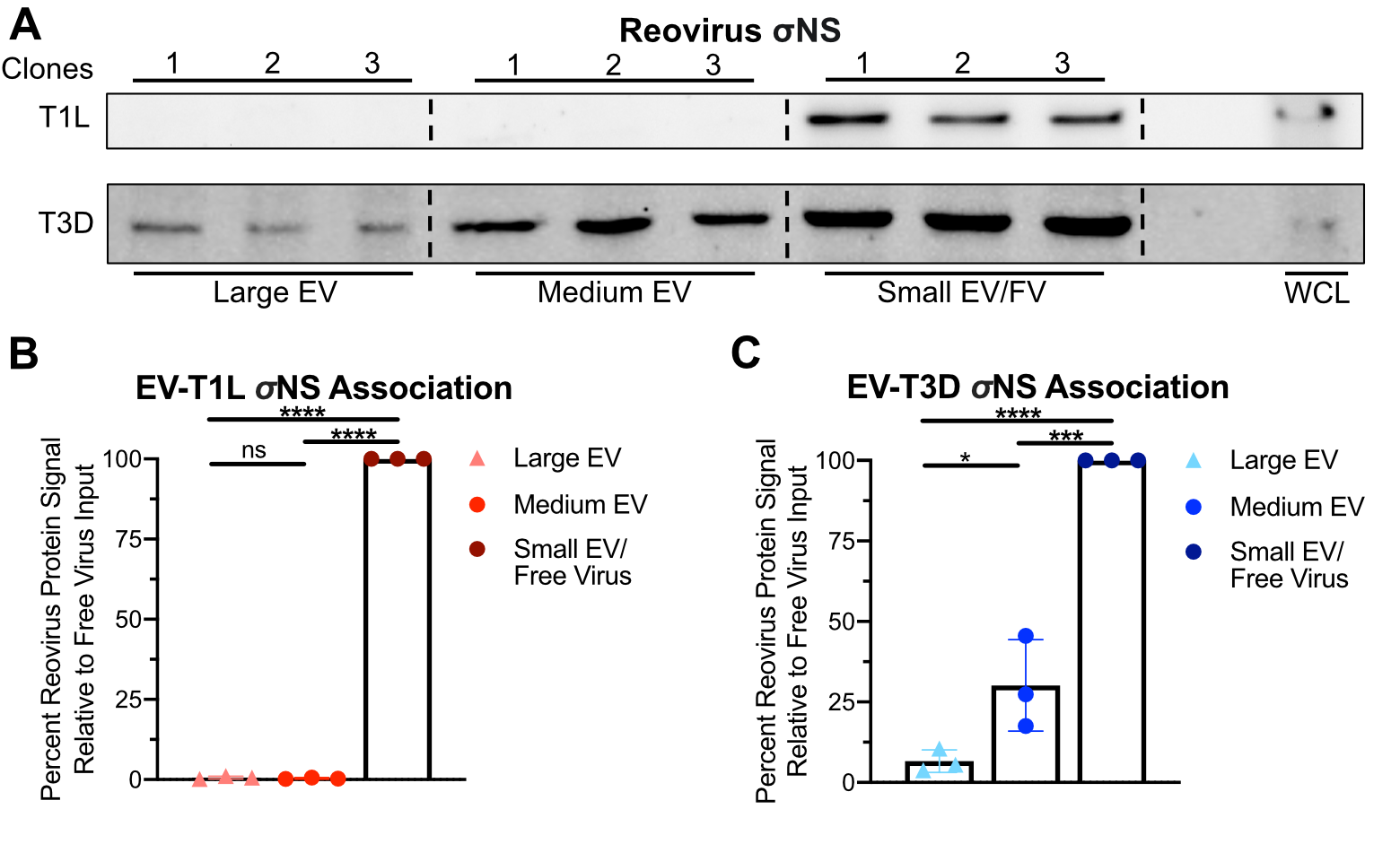

Supplement: S2 Fig — (A-C) L cells were adsorbed with three individual clones (C1-C3) of T1L or T3D reovirus at an MOI of 1 PFU/cell for 72 h. Reovirus nonstructural protein association with large EV, medium EV, and small EV/free virus fractions was quantified following SDS-PAGE and immunoblotting (A) for T1L σNS (B) or T3D σNS (C). Error bars indicate SD. n = 3. *, P < 0.05; ***, P < 0.001; ****, P < 0.0001 by one-way ANOVA with Tukey’s multiple comparisons prior to normalization. Protein signal was normalized as a percentage of maximum by dividing each adjusted volume value by the highest measured value for each clone within the blot. (TIF) [file ppat.1011637.s002.tif]

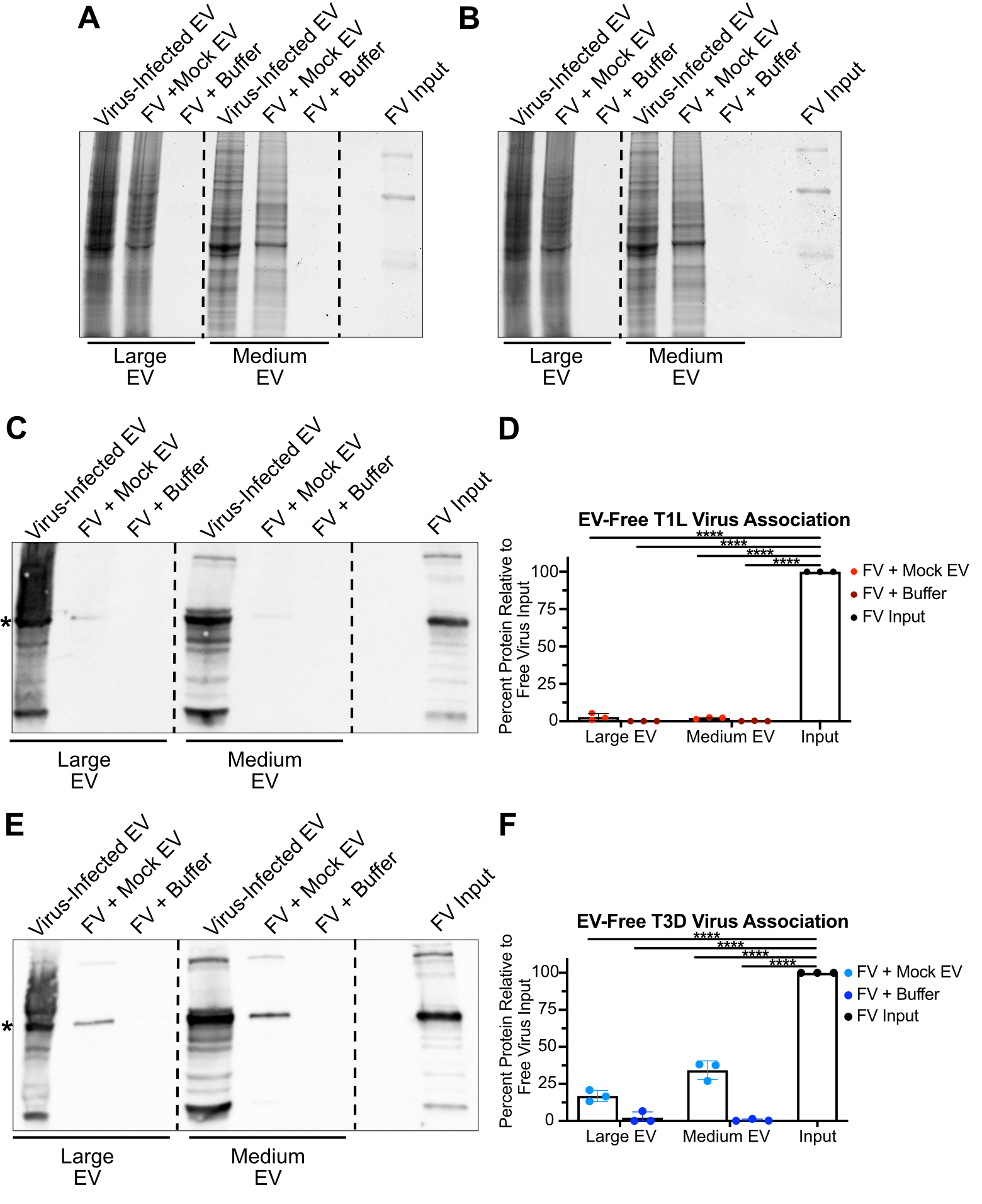

Supplement: S3 Fig — (A-F) L cells were adsorbed with three individual clones of T1L or T3D reovirus at an MOI of 1 PFU/cell. In parallel, triple the amount of L cells were adsorbed with medium (mock). After 72 h, large and medium EVs were harvested via centrifugation from reovirus-infected cells to constitute the “virus-infected EV” samples and from mock-infected cells. 1 x 10^9^ total PFU of free reovirus particles were mixed and incubated with large or medium EVs from mock-infected cells (mock EVs) or with EV storage buffer (buffer), then re-pelleted at respective centrifugation speeds. Equal volumes of all T1L (A, C, D) and T3D (B, E, F) samples were resolved by SDS-PAGE and Coomassie staining (A-B) or by SDS-PAGE with immunoblotting using anti-reovirus serum (C-F). The spontaneous association of free reovirus with mock large and medium EVs was quantified and compared to free T1L virus input (D) or free T3D virus input (F). Error bars indicate SD. n = 3. ****, P < 0.0001 by two-sample unpaired T test. (TIF) [file ppat.1011637.s003.tif]

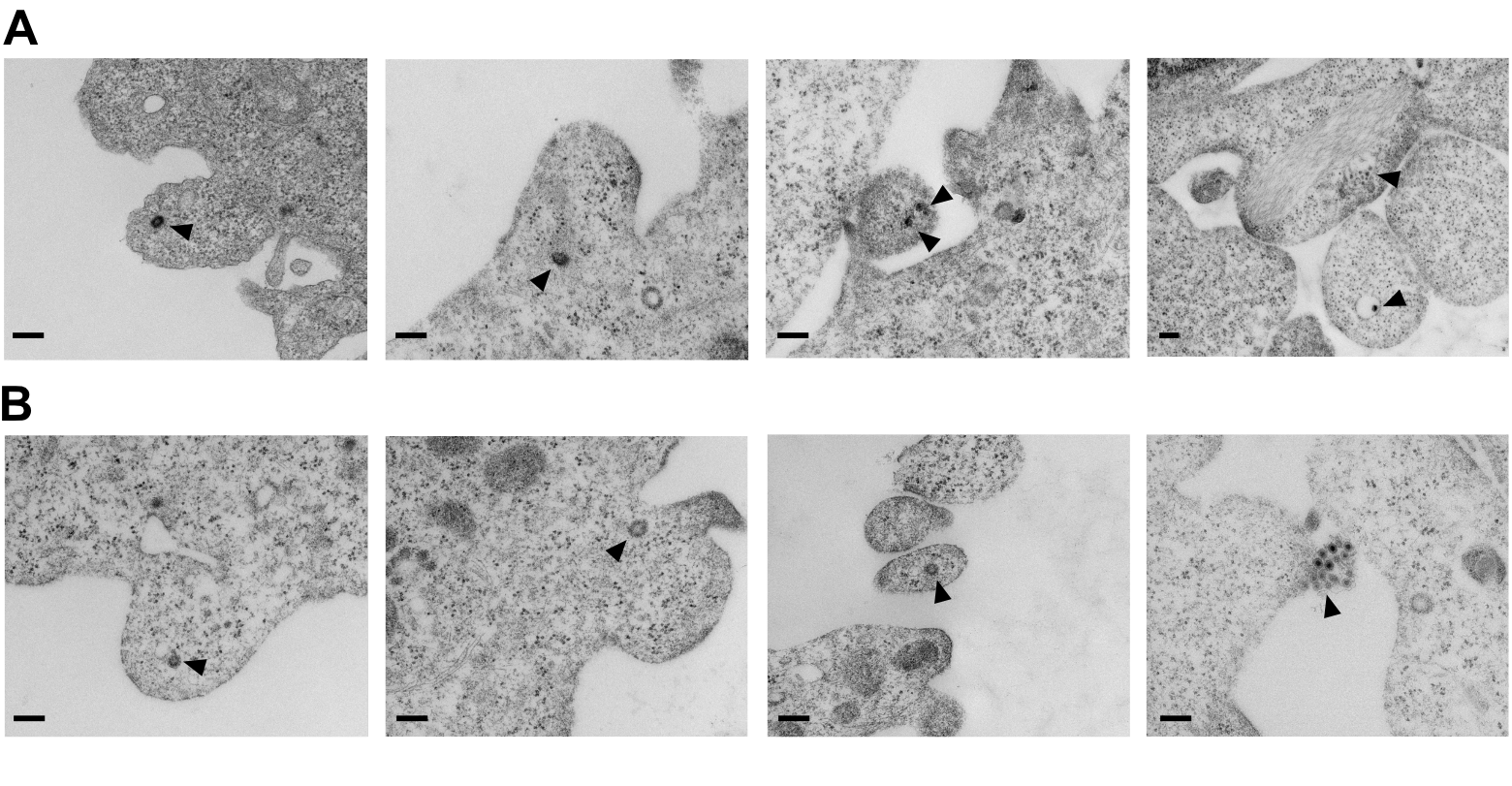

Supplement: S4 Fig — Transmission electron microscopy of T1L-infected (A) or T3D-infected (B) L cells at 24 h p.i. Arrows point to viral particles observed near bleb-like structures budding from the plasma membrane in or around cells. Scale bar = 200 nm. (TIF) [file ppat.1011637.s004.tif]

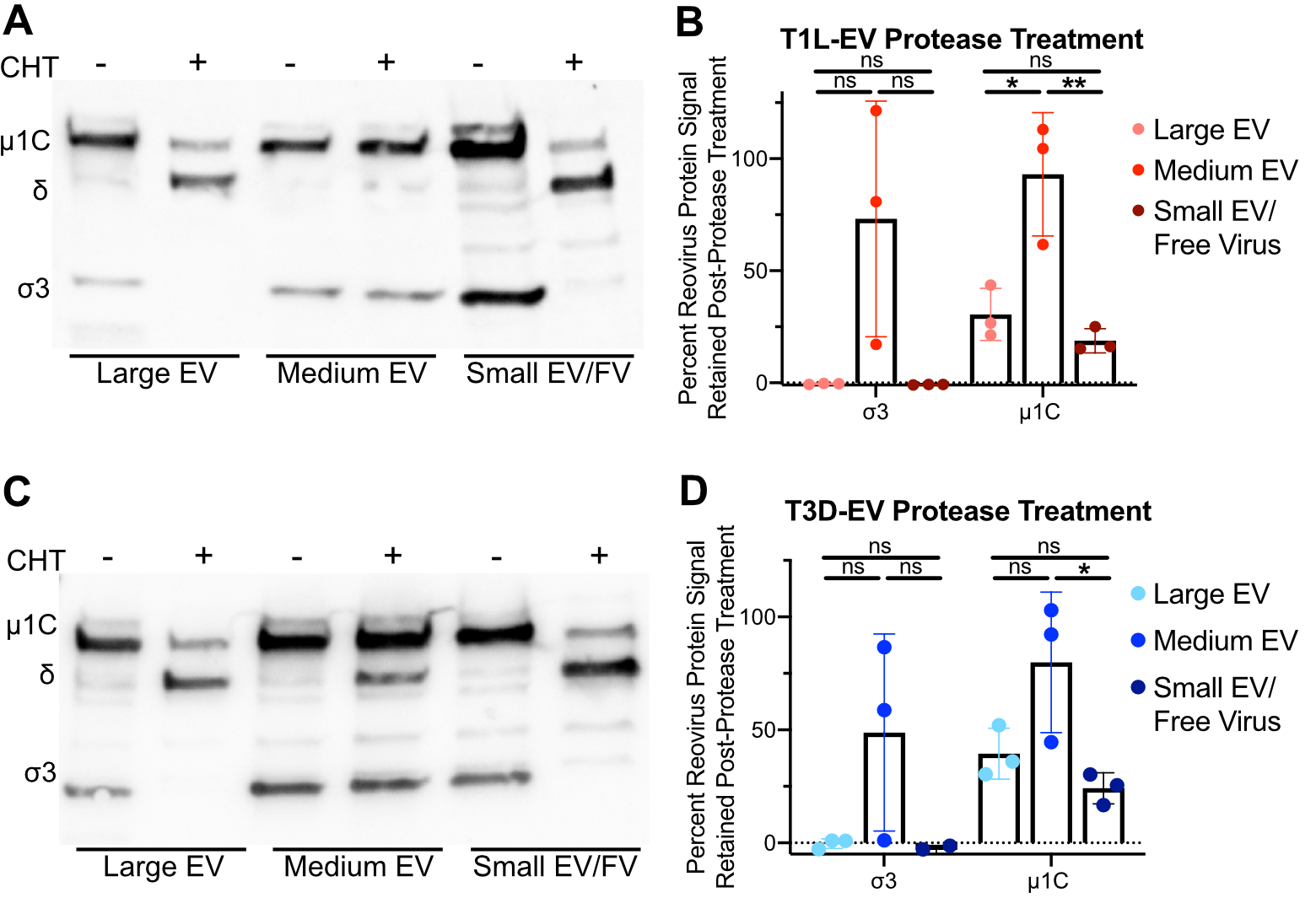

Supplement: S5 Fig — (A-D) L cells were adsorbed with three individual clones of T1L or T3D reovirus at an MOI of 1 PFU/cell for 72 h. Large, medium, and small EV/free virus fractions were harvested via centrifugation and each split into two aliquots containing equal volumes. One aliquot was left untreated (-), and the other aliquot was treated with 20 μg/mL of chymotrypsin (+). Reovirus T1L (A-B) and T3D (C-D) σ3 and μ1C proteins were visualized by and quantified by SDS-PAGE and immunoblotting using anti-reovirus serum. Shown are representative immunoblots for each virus strain alongside values quantified for the three clones. Error bars indicate SD. n = 3. *, P < 0.05; **, P < 0.01 by one-way ANOVA with Tukey’s multiple comparisons. (TIF) [file ppat.1011637.s005.tif]

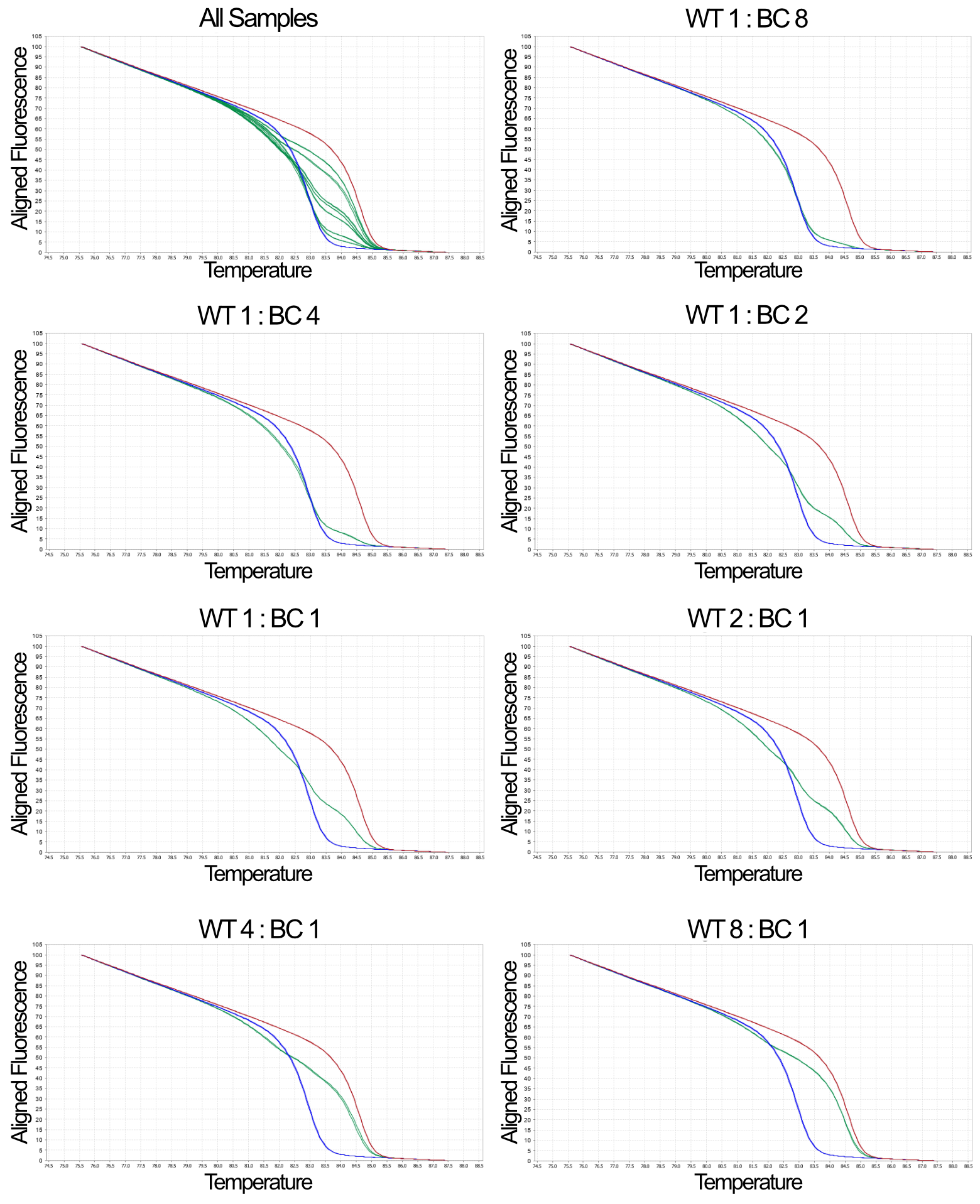

Supplement: S6 Fig — Normalized melt curves for control RNA from WT (red), BC (blue), and mixtures of WT and BC (green) at the indicated ratios are shown. (TIF) [file ppat.1011637.s006.tif]

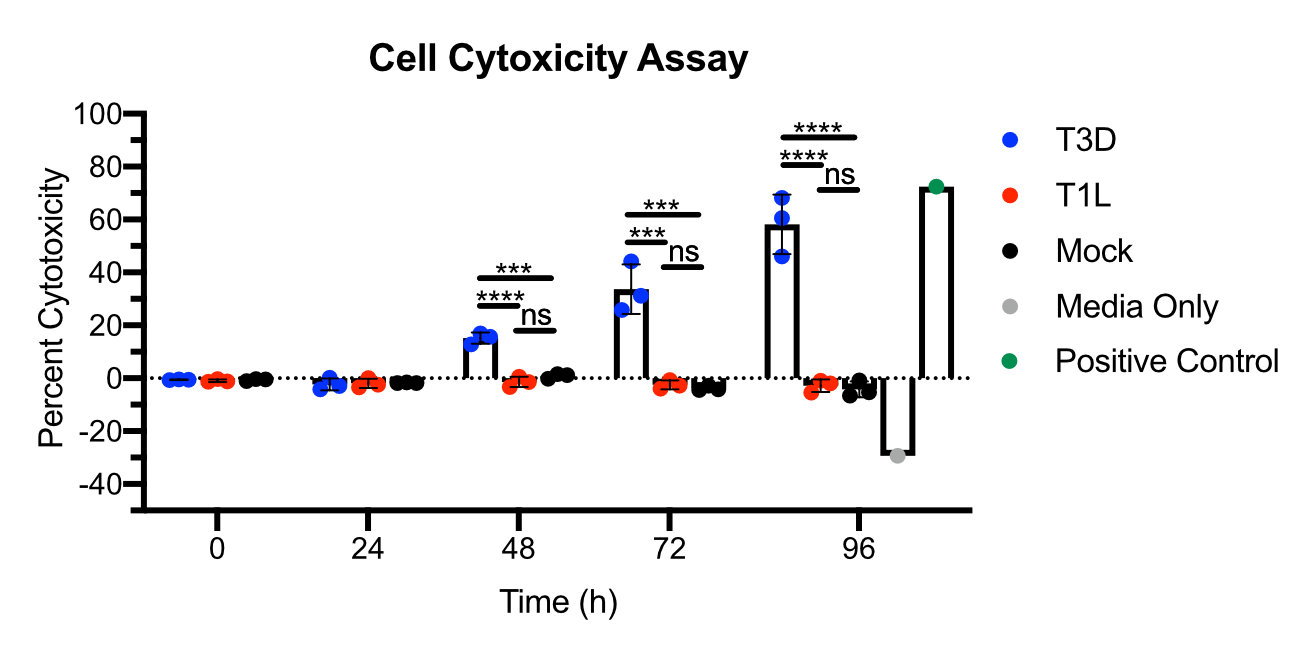

Supplement: S7 Fig — Caco-2 cells were adsorbed with medium (mock) or with three individual clones of T1L or T3D reovirus at an MOI of 5 PFU/cell. Cell membrane disruption was quantified for T1L-, T3D-, and mock-infected cells every 24 h for 96 h using an LDH assay. A medium-only negative control and a kit-specific positive control quantified in triplicate at 96 h are shown. Error bars indicate SD. n = 3. ***, P < 0.001; ****, P < 0.0001 by one-way ANOVA with Tukey’s multiple comparisons. (TIF) [file ppat.1011637.s007.tif]

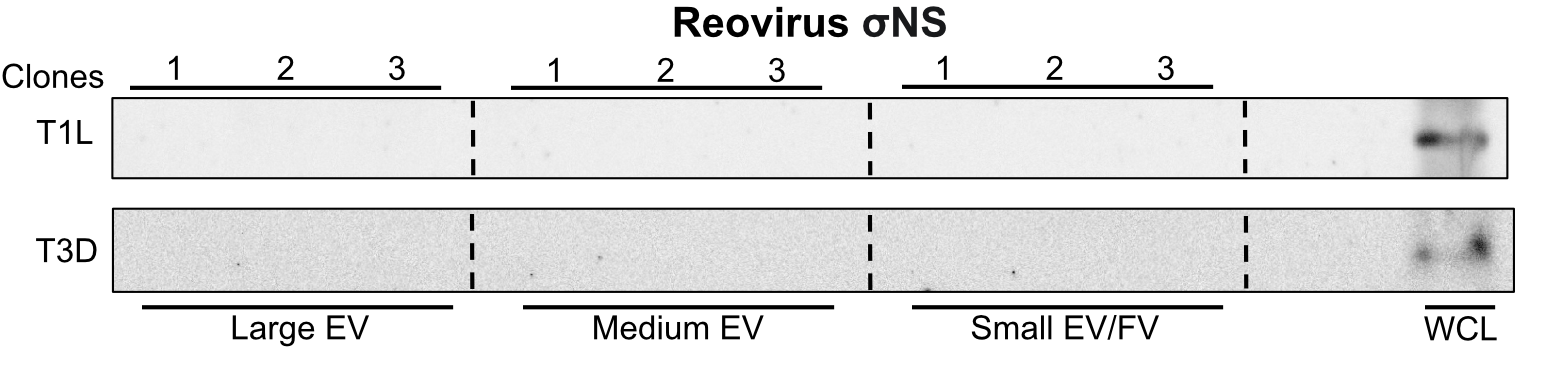

Supplement: S8 Fig — Caco-2 cells were adsorbed with three individual clones (C1-C3) of T1L or T3D reovirus at an MOI of 5 PFU/cell for 72 h. Reovirus nonstructural protein association with large EV, medium EV, and small EV/free virus fractions was quantified following SDS-PAGE and immunoblotting for T1L σNS or T3D σNS. (TIF) [file ppat.1011637.s008.tif]

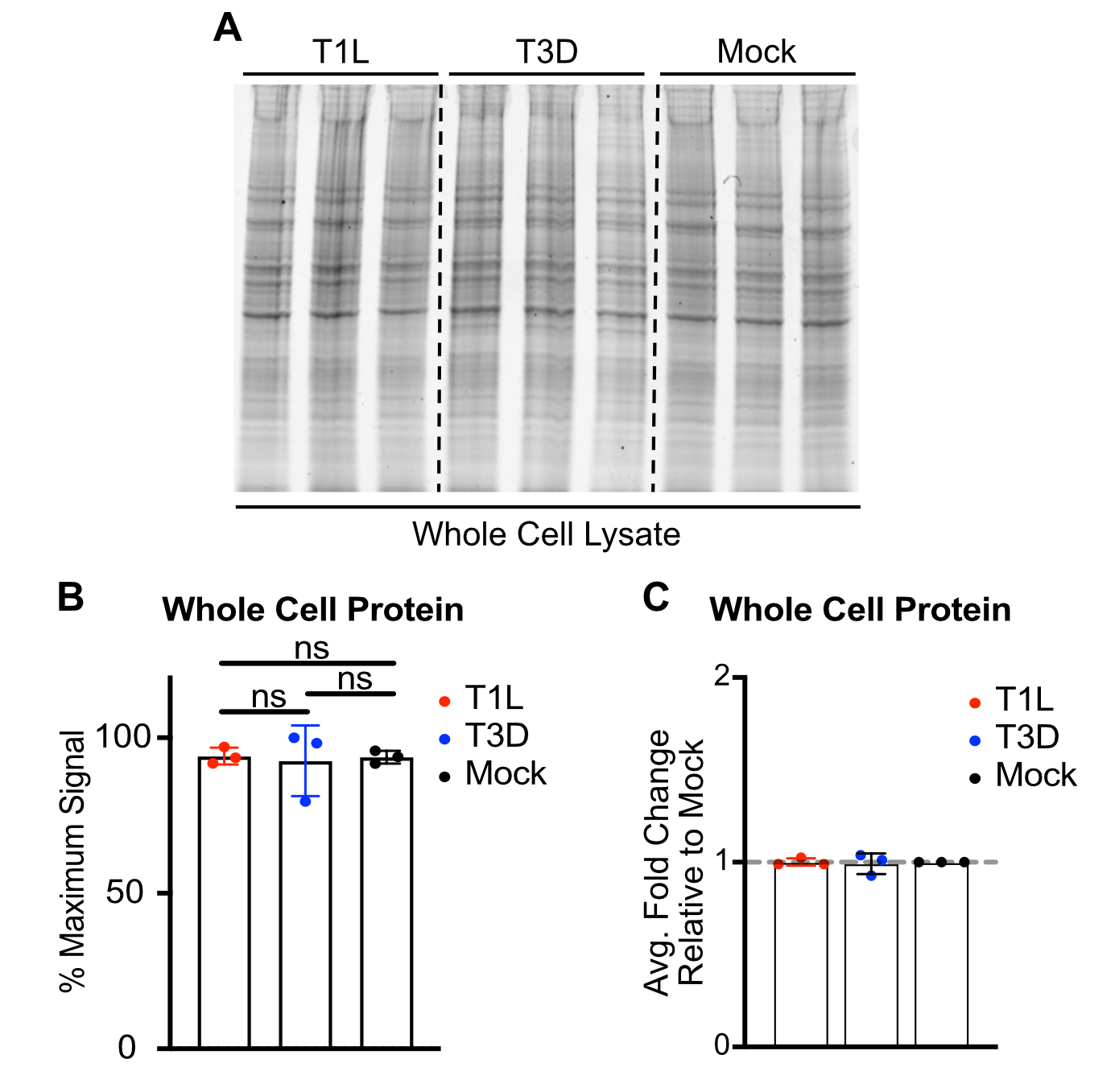

Supplement: S9 Fig — L cells were adsorbed with medium (mock) or with three individual clones of T1L or T3D reovirus at an MOI of 1 PFU/cell for 72 h. (A-C) Cells were lysed in RIPA buffer, and lysates were resolved by SDS-PAGE and Coomassie staining (A), three independent experiments were quantified (B), and normalized by dividing the average virus-infected value by the average mock-infected value (C). Error bars indicate SD, n = 3. Comparisons by one-way ANOVA with Tukey’s multiple comparisons. (TIF) [file ppat.1011637.s009.tif]

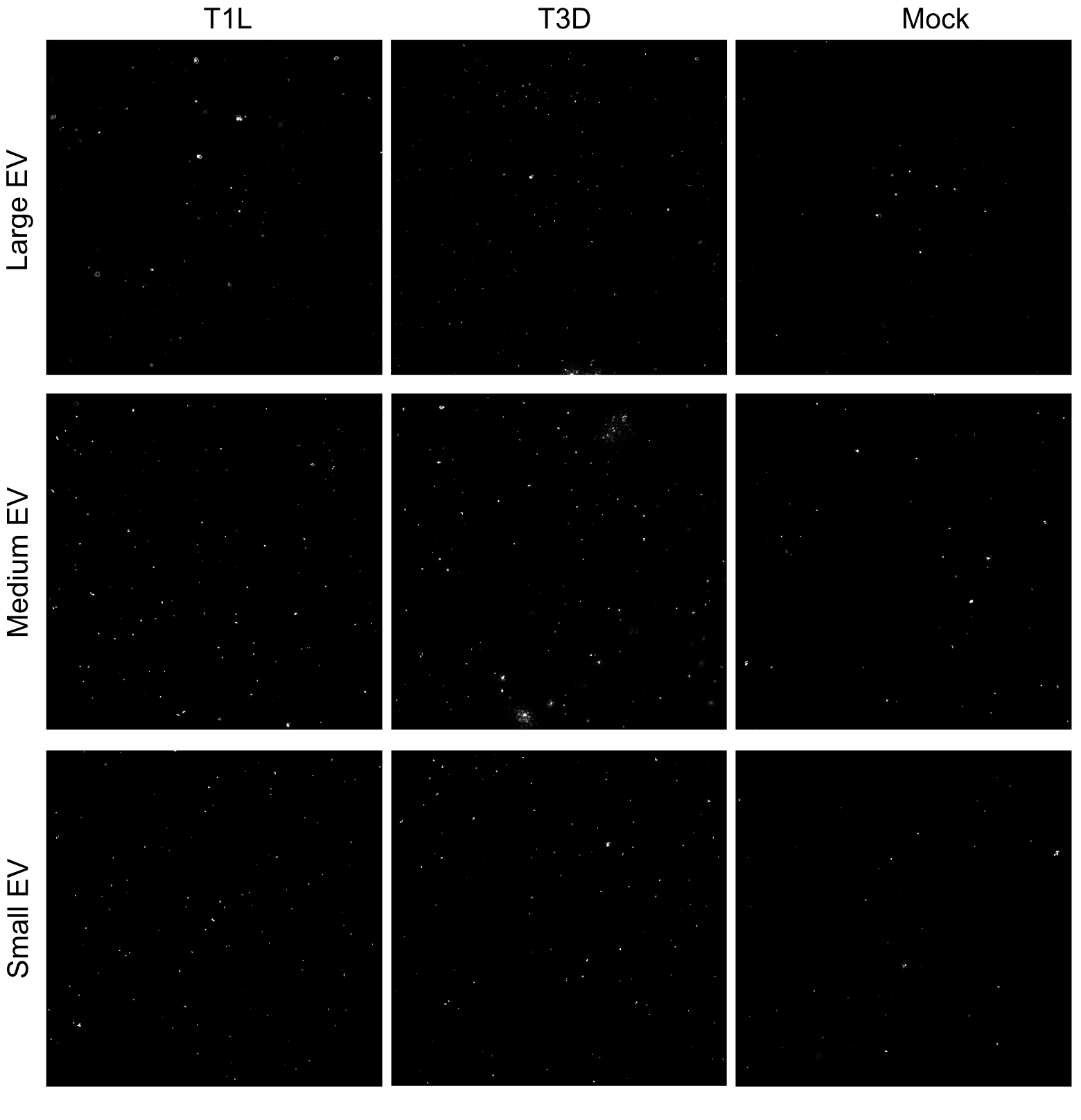

Supplement: S10 Fig — Representative confocal images described in Fig 6G–6H are displayed for a single field of view, which is made up of an 8 x 8 tile imaging structure under 63X oil immersion. (TIF) [file ppat.1011637.s010.tif]
