# Supplementary material for: Mammalian orthoreovirus can exit cells in extracellular vesicles
Source: PLoS Pathog. 2024 Jan 11;20(1):e1011637. doi: 10.1371/journal.ppat.1011637 (PMC10807757; doi:10.1371/journal.ppat.1011637)
Supplement: Attachment — Submitted filename: Smith_Reovirus Egress_Reviewer Response_FINAL2.docx [file ppat.1011637.s011.docx]

Dear Dr. Patton or Editor,

We thank each of our reviewers and editor for the helpful critiques of our manuscript PPATHOGENS-D-23-01432, which we think have significantly improved the conclusions presented in our revised study. We identified six main critiques from the reviewers to address: i) a request for a complementary technique to test the findings of our cell viability assay, ii) an inquiry into whether reovirus nonstructural protein associates with extracellular vesicle (EV) fractions, iii) a request for a revised discussion of our reovirus egress model, in which we more carefully consider the role of cell lysis in reovirus egress, iv) an explanation of the propensity for EV disruption during our negative-stain EM imaging procedure, v) an examination of the effect of mixing free reovirus particles with EVs, and vi) a request for a complementary technique to test the findings of our antibody-mediated neutralization protection assay of EV-associated reovirus.

We have addressed each reviewer critique in turn and have highlighted changes in the accompanying document. A brief overview of changes is provided here, and point-by-point responses are provided below, accompanying reviewer comments. **First**, to test the findings of our original trypan blue exclusion cell viability assay, we employed a fluorescence-based lactase dehydrogenase (LDH) assay, which quantifies LDH release into the culture medium upon damage to the plasma membrane. Similar to our original findings, we found that for both cell types included in the manuscript, T3D reovirus induces significantly more cellular cytotoxicity than T1L reovirus, which induces little cellular cytotoxicity above what we observe for mock-infected cells. **Second**, we employed SDS-PAGE and immunoblotting for reovirus nonstructural protein σNS to determine whether nonstructural reovirus proteins associate with EVs. We found that for L cell-derived fractions, T3D σNS protein associates with large, medium, and small EVs, while T1L σNS protein only associates with small EVs. In contrast, σNS from neither reovirus strain associated with Caco-2 cell-derived EV fractions. Potential implications of these discoveries are discussed. **Third**, we have updated the discussion of our model presented in Figure 7 to include the possibility that free T3D virus may egress from cells non-lytically as well as lytically. **Fourth**, we have described the possibility that EV disruption and consequent spilling of virus may have occurred during EV preparation for EM imaging. **Fifth,** we have assessed the propensity for reovirus particles to spontaneously associate with EVs by mixing EVs harvested from mock-infected cells with free virus particles. We found that free T1L virus does not significantly bind to large or medium EVs, while a proportion of free T3D virus does adhere to large and medium EVs; however, this spontaneous association is significantly less than the amount of free virus input into the interaction. **Finally,** we have employed a protease-based assay to test whether EVs protect reovirus from proteolytic cleavage. We observed partial protection of reovirus structural proteins for virus in the L cell-derived medium EV fraction but not the large or small EV fractions. These findings are consistent with results of antibody neutralization protection assays, in which reovirus in the L cell-derived medium EV fraction but not the large or small EV fractions was protected. Substantial text changes are highlighted in the revised manuscript, and six new supplementary figures have been added. We think results of the above experiments and text modifications strengthen the original conclusions of our study and in some cases provide new insights.

We again thank the reviewers for their thoughtful and detailed critiques of the manuscript, and we thank the editor and reviewers for consideration of the revised manuscript.

Best regards,

Kristen Ogden, Ph.D.

**Part I - Summary**

**Reviewer #1:**

The manuscript from Smith, Krystofiak and Ogden provides data supporting the hypothesis that mammalian reovirus exits from cells in extracellular vesicles. The authors provide data showing that reoviruses associate with small medium, and large EVs, but that only medium EVs provide protection from neutralization by antibodies, suggesting that the virions are protected within medium EVs, but not within small or large EVs. Further experiments show that small EV fractions contain free virus. The authors further describe that EV protection from neutralization is both strain and cell-type specific. This is a well-written, easy to follow paper. The experiments and data supporting the authors conclusions are for the most part robust. The findings will be of interest to the field and more generally within the virology community.

Major strengths include: (i) the experiments are rigorous and well-controlled. (ii) The data suggest that reovirus virions are released within medium EVs and that multiple particles can be contained with a single EV leading to more efficient infection. (iii) The phenotype of EV release is described in more than one cell type (L-cells and Caco-2 cells).

Weaknesses are: (i) Ultimately it remains unclear if reovirus particles are released within intact medium EVs and are thus protected from neutralizing antibodies OR if they are perhaps protected by some other mechanism(s). (ii) Although the experiments were carried out in two different cell types, they are both transformed cells and may not represent what would happen *in vivo*. (iii) No experiments were carried out to show that virions associated with EVs were more infectious than free virions. Experiments similar to those described by Chen et al. 2015. Cell 160:619–630 to compare the infectivity within EVs versus free virus would be strengthen the paper.

We thank you for your positive assessment of our manuscript and for pointing out specific areas for improvement. Although we cannot definitively conclude that reovirus particles are protected from neutralization because they are enclosed within medium EVs, it is currently the most likely explanation and is supported by the observation of particles that appear to be inside of medium EVs by negative-stain EM (Fig. 2), the protection from neutralization detected only in the medium EV fraction despite detection of virus particles associated with large EVs (Figs. 2 and 4B-E), and new data showing partial protection from proteolysis for only the medium EV fraction (S5Fig). Please see the “Major Issues: Key Experiments Required for Acceptance” section below for additional details. We agree with Points ii and iii above. Although we show that reovirus in EV fractions is infectious (Figs. 1H and 5F), we have not compared the relative infectivities of free and EV-associated reovirus. The proposed egress model that we present *in vitro* may not represent the egress mechanism of reovirus *in vivo*. As mentioned on Line 579-580, “…we do not yet know if this reovirus egress strategy occurs in humans or other animals….” We enthusiastically agree that infectivity studies similar to those described by Chen et al. would strengthen our current *in vitro* model, and we are actively working towards conducting studies in a newborn mouse model to compare the replication efficiency and disease severity of EV-associated and free reovirus. However, these studies fall outside of the scope of the current manuscript’s conclusions and will be reserved for a follow-up publication.

**Reviewer #2**:

Smith and colleagues present an elegant study investigating the role of extracellular vesicles in orthoreovirus egress. Mammalian orthoreovirus is a non-enveloped dsRNA virus with a segmented genome. Mechanisms of its egress are poorly understood. Much of the earlier literature has assumed that these viruses egress lytically, releasing from cells as free viral particles. A recent paper in 2020 provided evidence for these viruses egressing as free particles through a non-lytic mechanism: lysosomal exocytosis.

Here in this manuscript, the authors investigate the egress of two strains of mammalian orthoreovirus, T3D and T1L. These two strains apparently differ in their pathogenesism tropism and ability to induce apoptosis. According to the authors citation of previous literature, T3D induces apoptosis more efficiently than T1L.

Here the authors carry out a combination of experiments measuring plasma membrane permeability (with trypan), harvesting of different sizes of extracellular vesicles and quantification of associated virions, virion neutralization assays along with nice electron microscopy data. They conclude that both strains can be released in extracellular vesicles, protecting these virions from neutralization. Also they demonstrate that infection with these strains stimulates overall EV secretion from cells.

Overall this is a nice study that will be highly significant to the dsRNA virus community by highlighting the novel role of extracellular vesicles in the reovirus lifecycle.

A few suggestions for the authors that need clarification:

1. Figure 1B versus Figure 1D and 1F: It seems that for both strains a significant majority of the intracellular viral pools egresses by EVs at 48 hours ( and perhaps a bit earlier). By 48 hrs, the blots show almost equivalent amounts of virus of both strains has been released via medium EVs and small EVs/free virus ( see 1D and 1F). If anything T3D- the supposedly more lytic virus, has released medium EVs with virus quite early. . The difference in cell permeability at 48hrs between T3D and T1L is miniscule ( regardless of what the authors suggest with a significance asterisk)-by 72 hours, the amount of lysis in T3D population is ~50%, but there is no INCREASE in the level of T3D being released in small EV/free virus from 0-48 hrs, versus 0-72 hrs. Please explain.
2. How do the authors know that their vesicles did not lyse when they did the Ems? Likely that those viruses on the outside of the vesicles were once inside and during the EM procedure , the vesicle shrunk/broke and the viruses were released. Please indicate this possibility in the text if it cannot be ruled out. And indeed if it cannot be ruled out, then the authors data in Figures 3 and 4 and of course the cartoon need to be totally revised.

We are grateful for your positive evaluation of the strengths of our manuscript, and we thank you for specifying these needed improvements. We have addressed both Point 1 and Point 2 in the revised manuscript, and we detail the specific changes we have made in the “Major Issues: Key Experiments Required for Acceptance” section below.

**Reviewer #3**:

In this paper, Smith et al explore mechanisms by which mammalian reovirus exists host cells. While a specialized non-lytic exit pathway has been shown to occur in endothelial cells, whether this mechanism is universal is not understood. The authors report using two different reovirus strains that release of each of these strains from cultured fibroblasts and intestinal epithelial cells occurs independently of cell lysis. Instead, at least some proportion of the virus seems to exit cells in association with extracellular vesicles of various kinds. The remainder of the virus is released as free virus. When virus is associated with vesicles, virus seems resistant to neutralization. However, this neutralization efficiency varies with cell type from which the vesicles are derived and strain of infection. The authors demonstrate that multiple viruses can be delivered when associated with vesicles and therefore could launch an infection that allows for the possibility for reassortment.

The data presented in the manuscript are clear and support the authors’s conclusion that vesicle associated release of virus is one mechanism of reovirus release. The paper is also well written. It remains unclear how virus associates with extracellular vesicles and why there exist cell type and virus strain specific differences in the nature of association with extracellular vesicles. The authors could better characterize whether the virus is inside or outside the vesicles (or what proportion is where), whether vesicle association is an active process or an artefact of purification or simply a passive effect of virus-membrane association. Finally, it appears that even when the particles are vesicle associated, at least half the virus is released also as free virus. Thus, the relevance of this mode of release and transmission between cells remains unknown.

Thank you for your assessment of the strengths and limitations of our findings. We agree with your points above—we too are interested in the relevance of EV-associated reovirus egress and transmission between cells and question whether our *in vitro* findings will be recapitulated in an *in vivo* model. We plan to build on our current work with a future publication centering around *in vivo* infectivity studies. We plan to address the additional gaps in knowledge you mention in follow-up studies, including i) identifying the EV biogenesis pathways directly involved in reovirus-EV egress and ii) uncovering mechanisms of the observed cell type- and virus strain-specific differences in reovirus-EV association. For the current revised manuscript, we have included additional experiments to address two of your above points, which we think are important for supporting the conclusions of our study. These involve better characterizing whether reovirus is packaged internally or bound externally on EVs of interest and whether our observed reovirus-EV association is an artefact of spontaneous aggregation of virus with membranous structures. We detail the specific experiments we have included to address both points in the “Major Issues: Key Experiments Required for Acceptance” section below.

**Part II – Major Issues: Key Experiments Required for Acceptance**

**Reviewer #1**:

1. The authors use trypan-blue exclusion as a means to determine membrane disruption. The disruption of cell membranes by viruses is likely not all-or-none, but rather is a continuum – the reovirus μ1 protein is a potential viroporin and the relative amounts of free μ1 may determine the degree of membrane permeability. The trypan-blue exclusion assay is less sensitive than similar assays that use fluorescent dye uptake (perhaps combined with flow cytometry) and is necessarily subjective using the methods described. Trypan-blue can also lead to direct effects on dead cells that lead to them rupturing and having a dim uptake of dye. The concern would be that potentially membrane disruption is occurring in the T1L-infected cells, but the level is below the threshold of sensitivity for the Trypan-blue assay. This may be important as membrane disruption does occur in living cells and may be a driver of EV generation – budding or blebbing of membranes that contain wounds may generate EVs that are not intact. It would be interesting to compare the results from Trypan-blue with perhaps propidium iodide staining.

Thank you. In the past, we have had challenges obtaining consistent and reliable results using propidium iodide staining with flow cytometry. However, we agree that a more sensitive and less subjective assay assessing cell membrane damage than trypan blue staining could strengthen our conclusions. We employed a fluorescence-based lactate dehydrogenase (LDH) assay. This assay uses a machine-based readout to measure the level of cytosolic enzyme LDH released from cells upon membrane damage. We infected L cell and Caco-2 cell monolayers with T3D, T1L, or mock-infected with media. Every 24 h for a total of 96 h, we analyzed each condition. As with the trypan blue exclusion assay, the LDH assay showed that for both L cells and for Caco-2 cells, T3D reovirus induces significantly more cellular cytotoxicity than T1L reovirus, which does not induce cellular cytotoxicity much above what we observe for mock-infected cells. We have included text and accompanying figures explaining this additional observation in Lines 147-150, Lines 356-358, Supplemental Figure 1, and Supplemental Figure 7.

2. The authors show nicely that viral structural proteins associate with large, medium, and small EVs. However, it would be interesting to see if nonstructural proteins are also present, e.g., μNS or σNS. This may be pertinent, as it is feasible that virions buried within remnants of viral factories may be protected from neutralizing antibodies, despite the fact they are not contained inside an EV. Excluding the presence of nonstructural proteins from EVs would remove this concern.

Thank you for suggesting this interesting line of inquiry. To assess whether reovirus nonstructural protein associates with each of our EV fractions, we infected L cell monolayers with T3D and T1L for 72 h. We used sequential differential centrifugation to harvest large, medium, and small EV/free virus fractions, and then employed SDS-PAGE and immunoblotting for reovirus nonstructural protein σNS, a key component of cytoplasmic reovirus factories. We found that for L cell-derived fractions, σNS protein association is strain-dependent; T3D σNS protein associates with large, medium, and small EVs. T1L σNS protein, on the other hand, only associates with small EVs. There appear to be further cell type-specific differences in this association phenotype, as σNS protein from neither reovirus strain was detected in any Caco-2 cell-derived EV fraction. There are multiple potential explanations for differences in σNS association with EV fractions derived from T3D- versus T1L-infected cells. First, T3D induces apoptosis more efficiently than T1L (Figs. 1B and 5C, S1 and S7 Figs). Apoptotic blebs vary broadly in size and may be detected in any EV-enriched fraction. Signal from σNS detected in large and medium EV fractions from L cells may primarily be derived from pieces of virus factories incorporated in apoptotic blebs, which would explain their absence in corresponding T1L fractions. While T3D forms globular factories, T1L forms filamentous factories that associate with microtubules. Factory morphology and host protein interactions also may alter σNS uptake into released EVs, particularly large and medium EVs released from the plasma membrane. In either case, if T3D particles are packaged into the same EVs as σNS, they could potentially be protected from the environment by nonstructural proteins. However, this mechanism does not explain the protection of T1L in medium EVs derived from L cells or Caco-2 cells (Figs. 4B-C and 5G-H, S2 and S8 Figs). Reasons for the lack of σNS presence in T3D-infected Caco-2 cell-derived large and medium EV fractions are unclear but could correlate with differences in apoptosis pathways. The σNS protein can bind cellular RNA. RNA binding, protein misfolding, or ubiquitinylation and sorting by ESCRT complex proteins could serve as a mechanism to load T1L or T3D σNS into small EVs, which are known to package RNA and protein cargo. Although exosome cargo can differ by cell type, it is unclear why σNS is present in reovirus-infected L cell-derived small EV fractions but not in any T1L- or T3D-infected Caco-2 cell-derived EV fractions (S2 and S8 Figs). We have included text with discussion and accompanying figures explaining these observations in Lines 179-187, Lines 363-365, Lines 506-529, Supplemental Figure 2, and Supplemental Figure 8.

**Reviewer #2:**

1. Figure 1B versus Figure 1D and 1F: It seems that for both strains a significant majority of the intracellular viral pools egresses by EVs at 48 hours ( and perhaps a bit earlier). By 48 hrs, the blots show almost equivalent amounts of virus of both strains has been released via medium EVs and small EVs/free virus ( see 1D and 1F). If anything T3D- the supposedly more lytic virus, has released medium EVs with virus quite early. The difference in cell permeability at 48hrs between T3D and T1L is miniscule (regardless of what the authors suggest with a significance asterisk)-by 72 hours, the amount of lysis in T3D population is ~50%, but there is no INCREASE in the level of T3D being released in small EV/free virus from 0-48 hrs, versus 0-72 hrs. Please explain.

Thank you for bringing this to our attention. We agree with your point that we do not see evidence of membrane disruption induced by T3D at 48 h p.i., yet we do observe evidence of T3D protein association, primarily in the free virus fraction but also in association with medium EVs, at 48 h p.i. Our model indicated that T3D requires cellular lysis for free virus to egress. We concede that this may not be wholly correct, and we lack direct evidence to support the claim. In the revised manuscript, we have updated the discussion of our model (Fig. 7) in Lines 454-457 to include the possibility that free T3D virus may egress from cells non-lytically as well as lytically. We have also clarified a related point in Lines 136-140 to indicate that the graph in Fig. 1A displays the quantification of total virus following freeze-thaw of the monolayer and medium, not solely released virus. For T3D, we do observe an increase in viral protein associated with EVs and as free virus when we compare the 48 h p.i. timepoint to the 72 h p.i. timepoint (Fig. 1F-G). However, the total infectious virus present inside of infected cells and in the medium does not appear to increase during this time period (Fig. 1A).

2. How do the authors know that their vesicles did not lyse when they did the Ems? Likely that those viruses on the outside of the vesicles were once inside and during the EM procedure , the vesicle shrunk/broke and the viruses were released. Please indicate this possibility in the text if it cannot be ruled out. And indeed if it cannot be ruled out, then the authors data in Figures 3 and 4 and of course the cartoon need to be totally revised.

Thank you for bringing this important point to our attention. We cannot exclude the possibility that some fraction of EVs were disrupted and consequently spilled virus during preparation for EM imaging. We have included this possibility in Lines 218-220. We also readily concede that we don’t know with certainty whether the virus particles are enclosed within the vesicles. We mentioned this lingering question in our original manuscript and in the revised manuscript in Lines 223-224. However, Dr. Krystofiak is experienced at imaging EVs, having worked with several faculty members of the Vanderbilt Center for Extracellular Vesicle Research to do so, and in our EM images we see many intact EVs of appropriate size. In addition to neutralization protection assays, we have now conducted protease protection assays, which both support the idea that medium EVs but not large or small EVs shield reovirus particles from the environment (Figure 4, Figure 5, and Supplemental Figure 5). We concede that there is a possibility that a mechanism other than membrane enclosure mediates this phenotype, and discuss this possibility (e.g., Lines 519-520) but current evidence is consistent with vesicle enclosure of at least a subset of the particles in the medium EV fraction. Thus, we do not think we need to revise Figures 3 and 4 or our model.

**Reviewer #3:**

1. Since extracellular vesicles can be produced from non-infected cells, what is the effect of mixing purified virus with extracellular vesicles? Can the authors demonstrate that virus will not associate with membranes under these conditions? This speaks to the specificity and mechanism of membrane interactions.

Thank you for suggesting this important line of questioning. We have addressed your questions by assessing the propensity for reovirus particles to spontaneously associate with EVs by mixing EVs harvested from mock-infected L cells with free reovirus particles. We found that free T1L virus does not significantly adhere and co-pellet with large or medium EVs, while 20-30% of input T3D virus adheres and co-pellets with large and medium EVs; however, this spontaneous association is significantly less than the amount of free virus input into the reaction, and it appears relatively smaller than the amount of T3D released in association with EVs during infection. The more efficient association of T3D with large and medium EVs from mock-infected cells compared with that of T1L is consistent with its apparently more efficient binding to L cells. Thus, we think nonspecific virus adhesion to large and medium EVs is unlikely to fully explain the association of these particles with the EVs during egress. We have included text and accompanying figures explaining these observations in Lines 249-271, Lines 432-434, and Supplemental Figure 3.

2. Can the authors use techniques other than neutralization to demonstrate that virus is present inside of the vesicles? Perhaps accessibility to a protease? If the virus is not neutralized by s1 specific sera, will it infect cells that lack s1 receptors? In other words, how does vesicle associated virus launch infection? What other antisera against reovirus capsids fail to neutralize virus? In conditions where vesicle associated virus was not protected from neutralization, were other antibody concentrations used? Can neutralization resistant virus become sensitive upon solubilization/disruption of the membrane?

Thank you for detailing such thoughtful questions. The questions you have posed regarding EV-associated reovirus receptor usage and infection initiation are of interest to us, and we are excited to begin answering them in future studies. Because the methods required to address these questions are highly complex, and many of the questions fall outside the scope of the current study, we must reserve many of them for our future work. However, we sought to answer the first two questions you posed, which are directly related to and could support conclusions of the current study. To test whether EV association provides a protective effect to reovirus under different assay conditions, we devised a protease protection assay. We adsorbed L cells with T3D or T1L reovirus, and after 72 h, we harvested large, medium, and small EV/free virus fractions. We treated the fractions with 20 μg/mL chymotrypsin, which converts reovirus virions to infectious subvirion particles through a well-studied series of proteolytic cleavage events. Then, we resolved the viral proteins by SDS-PAGE and immunoblotting and quantified two cleavage/conversion events. We observed partial and significant protection of reovirus structural proteins from proteolysis when associated with the medium EV fraction. These results are consistent with results of the neutralization protection assay and support the idea that medium EVs enclose and protect reovirus particles from the environment. We have included text and accompanying figures explaining these observations in Lines 303-319 and Supplemental Figure 5.

You asked several questions about antibody specificity and concentration. Plaque reduction-based neutralization assays are time consuming and low throughput. Although there are many additional questions we hope to address, we have presently focused on gathering evidence that emphasizes the differences in protection we observe for reovirus associated with the medium EV fraction and other EV fractions. Initially, we tested two concentrations of a σ1-specific monoclonal antibody and did not detect neutralization for any fraction or for control virus. We also tested a range of ten-fold dilutions of polyclonal reovirus antiserum and began to detect neutralization at a 1:1,000 dilution. However, a consistent 2-3 log neutralization of free virus was detected using a 1:100 dilution. Thus, we chose this as our working antiserum dilution. We have not yet tested antiserum concentrations higher than 1:100 or additional concentrations of monoclonal antibody, and we have not tried to determine whether there is an unprotected virus population in the 10K fraction or if there are neutralization-resistant populations in any fraction or for free reovirus. While we hope to conduct such studies in the future, there will be limits to the antiserum concentrations and levels of neutralization we can achieve, and we do not think these experiments are necessary to support the conclusions we make in the current manuscript.

3. Does the spread of virus in a population where infection is initiated at low MOI dependent on EV-associated virus? What is the consequence of blocking extracellular formation. Are inhibitors of this process effective at reducing virus release or cell-to-cell spread, or coinfection efficiency? Addressing this type of question will address the relevance of this exit mechanism.

We appreciate the forward-looking nature of your critique. We enthusiastically agree that more firmly addressing the relevance of our proposed reovirus egress mechanism, potentially through studies assessing viral spread or investigating the consequence of inhibiting EV formation, would be highly informative. We agree that these are important gaps in knowledge that must be addressed; however, the questions you ask here are highly complex. Because we do not yet understand the EV biogenesis pathway that reovirus interacts with to induce EV-associated release, answering your inquiries would involve inhibiting numerous EV pathways, which is likely to have a negative impact on cell viability. In each case, optimization of inhibitors and their concentrations would be required. Studying viral spread would likely also be complex. Although we can control the input into an infection (EV-associated or free virus), after a single round of infection, multiple forms of virus would be released. Thus, these studies also likely would require the use of EV biogenesis inhibitors in addition to specialized imaging systems that we do not yet have in place. To stay within the scope of the current study, we targeted the most relevant gaps in knowledge, including virus adhesion to different sizes of EVs and EV-mediated protease protection of EV-associated virus. We hope to answer several of your queries with more nuanced and mechanistic approaches in future manuscripts.

**Part III – Minor Issues: Editorial and Data Presentation Modifications**

**Reviewer #1:**

1. The particles shown in Figure 2 in the Large and medium EVs differ in morphology – it looks like there are some empty particles and some full particles and other particles differ again. It would be helpful to indicate in the Figure legend what the arrowheads indicate, but also it may be interesting to classify the different types of particles that are present in the large versus medium EVs.

Thank you for calling our attention to this point. We have specified in the Figure 2 legend that arrowheads indicate virus particles. While we agree that the classification of different types of virus particles, such as empty and full, may yield interesting data about virus egress pathways, it is likely that many virus particles are hidden from view internally or on the “backside” of electron-dense EVs, which often are too thick for our TEM microscope to fully visualize. Addressing your query to its full extent would likely require thin sectioning a population of large and medium EVs, so that we can clearly visualize the entire contents of an EV as well as any externally bound particles or disrupting EVs with detergent and visualizing and quantifying the contents. We will keep this idea in mind for a follow-up publication.

2. Line 135, the authors state that the initial cell binding varied between the two strains. Please provide the data or a reference

Thank you for bringing this to our attention. While there is no direct evidence for a binding difference for T1L and T3D, we inferred such a difference based on the use of an identical input of 1 PFU/cell for each virus and detection of an ~2 log difference in titer at the 0 h time point, after adsorption and washing of the cell monolayer. We have edited the text in the revised manuscript on Lines 136-140 to reflect the source of this inference.

**Reviewer #2:**

1. Paper can benefit from editing and streamlining the figures.

Thank you for your evaluation. To represent our findings effectively and transparently, in many cases we included an example of “raw data” alongside a panel showing quantified replicates, including individual data points. Although this format can make figures somewhat “busy,” we want the data to accurately reflect the variability we observe among EV preparations. For EM studies, we show more than a single image of each condition to reflect the diversity of outcomes more accurately.
